# Supplementary material for: The Working Environment in Primary Healthcare Outpatient Facilities: Assessment of Physical Factors and Health Professionals’ Perceptions of Working Environment Conditions
Source: Int J Environ Res Public Health. 2024 Jun 28;21(7):847. doi: 10.3390/ijerph21070847 (PMC11276708; doi:10.3390/ijerph21070847)
Supplement: Supplementary file 1 [file ijerph-21-00847-s001.zip › ijerph-2963485-supplementary.pdf]

Supplementary Materials

The working environment in Primary Healthcare outpatient facilities: assessment of physical factors and health professionals' perceptions of working environment conditions

Marta Regina Cezar-Vaz, Clarice Alves Bonow, Joana Cezar Vaz, Carlos Henrique Cardona Nery, Mara Regina Santos da Silva, Flávia Santana Freitas, Aline Soares Alves, Daniela Menezes Galvão, Joice Simionato Vettorello, Jociel Lima de Souza and Joaquim Vaz

The physical factors monitored indoors (rooms) and outdoors were measured at specific points or locations, as shown in Figures S1 and S2.

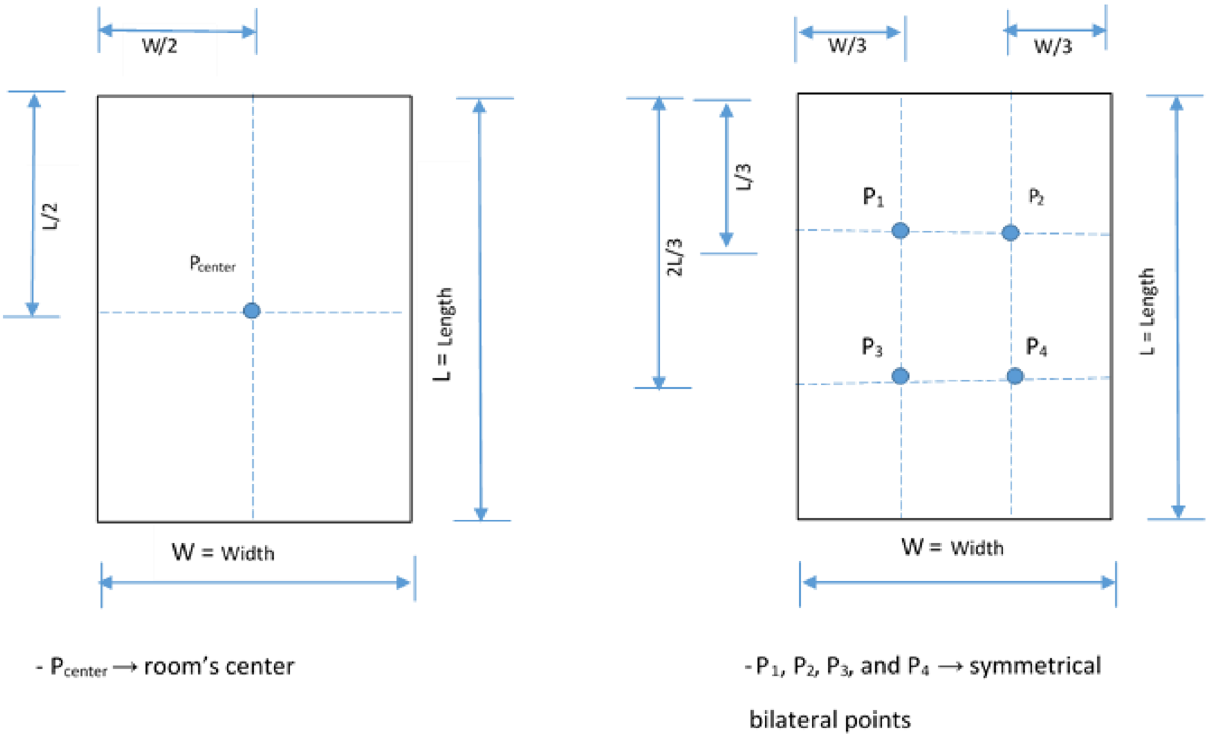

Figure S1 – Reference points for indoor measurements

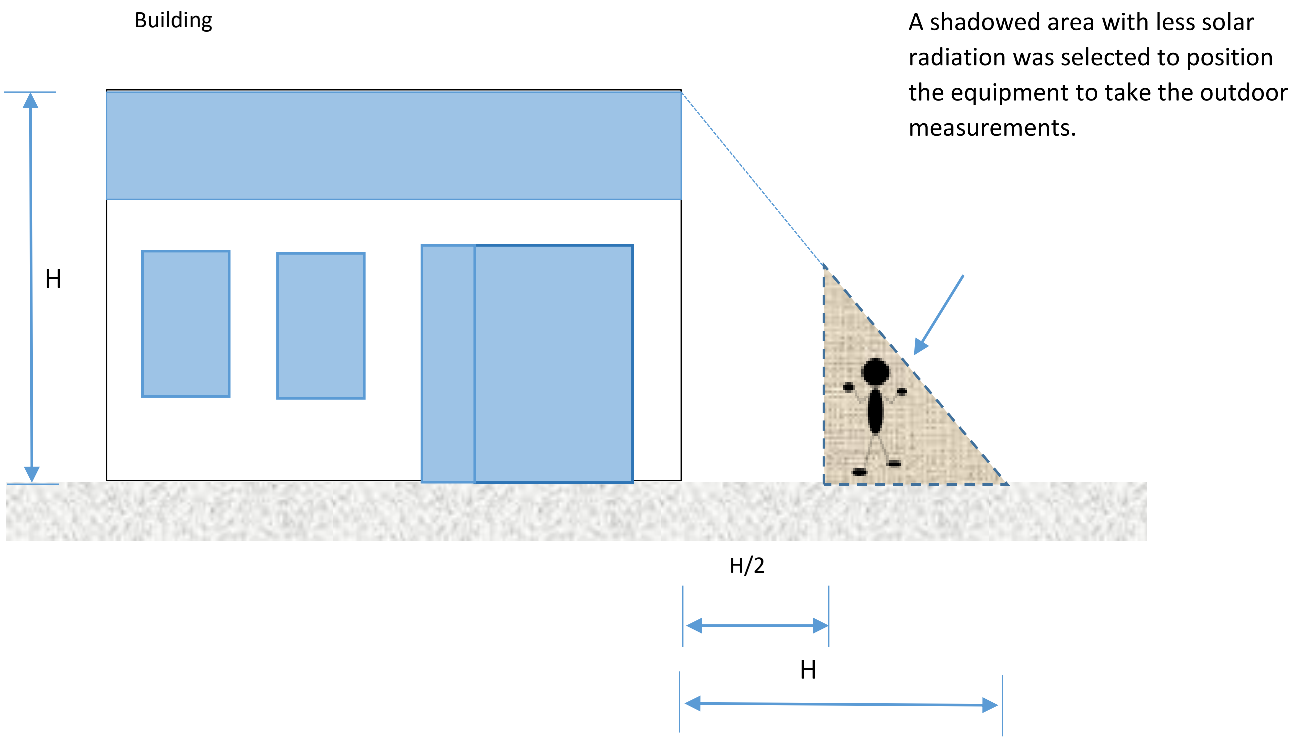

Figure S2 – Illustration of a shadowed area used in the outdoor measurements

Table S1. Characterization of the sample of professionals working in the PHC facilities selected for the environmental assessment

**Variables**	n=210<br>n (%)
Age (years)*	42.4 ± 9.9
Sex	
Male	32 (15.3)
Female	177 (84.7)
Race	
Caucasian	162 (77.9)
Afro-descent	22 (10.6)
Mixed race	24 (11.5)
Marital Status	
Single	59 (28.1)
Married/Consensual union/	122 (58.1)
Separated/Divorced/	24 (11.4)
Widowed	5 (2.4)
Education	
Up to High School	84 (40.0)
Some undergraduate studies/bachelor's degree/Technician	84 (40.0)
Specialization/Master's degree/ Doctoral Degree	42 (20.0)
Number of children **	1 (1 – 2)
BMI (kg/m^2)*	28.9 ± 5.9
Monthly income (minimum wage)	
Up to 2 times the MW	84 (41.0)
2 to 4 times the MW	64 (31.2)
> 4 times the MW	57 (27.8)
Second job	33 (15.7)
Profession	
Nurse	25 (11.9)
Physician	25 (11.9)
Nursing technician/Aide	43 (20.5)
Community health agent	100 (47.6)
Dentist	7 (3.3)
Oral technician/Aide	8 (3.8)
Other	2 (1.0)
Working experience (years)**	11.5 (5 – 17.3)
Working time in the PHC service (years)**	9.5 (1.1 – 14.4)
Weekly working hours*	42.9 ± 11.2
PHC shift work	
Daytime	196 (93.8)
Nighttime	4 (1.9)
Nighttime/daytime	6 (2.9)
Other	3 (1.4)

* described by ± SD; ** described by median (percentiles 25-75)

Table S2. Additional material (condensed information from health professionals in the PHC facilities analyzed, in %, incorporated into the environmental conditions database of the 23 PHC facilities)

23 APS facilities (in Portuguese UBS)	**Barrier perception**	**Facilitator perception**	**Physical risk perception**	**Uncomfortable room temperature**	**Annoying and irritating noise**	**Poor lighting**	**Lack of ventilation in the environments**	**Moderate/severe barrier perception**	**Moderate/severe facilitator perception**	**Moderate/severe physical risk perception**
1	91.7	66.7	75	66.7	0	0	16.7	58.3	50	66.7
2	100.0	66.7	100	88.9	22.2	33.3	55.6	100	33.3	77.8
3	100.0	58.8	88.2	64.7	41.2	17.6	41.2	88.2	47	82.3
4	100.0	87.5	100	100	37.5	100	62.5	87.5	25	75
5	66.7	83.3	100	50	33.3	0	0	0	83.3	50
6	100.0	75	91.7	83.3	41.7	58.3	25	66.7	41.7	75
7	100.0	93.3	100	86.7	26.7	13.3	13.3	86.7	66.7	73.3
8	88.9	88.9	88.9	66.7	33.3	11.1	0	66.7	66.7	77.8
9	50.0	100	75	75	0	0	0	25	75	25
10	75.0	87.5	87.5	50	0	0	0	75	50	62.5
11	100.0	100	100	66.7	0	33.3	33.3	100	66.7	100
12	100.0	33.3	100	66.7	0	0	33.3	100	33.3	33.3
13	84.6	92.3	100	69.2	23.1	7.7	15.4	46.2	77	53.8
14	87.5	62.5	100	87.5	37.5	37.5	25	75	37.5	75
15	100.0	60	80	80	70	30	40	90	20	70
16	85.7	92.9	92.9	38.5	15.4	15.4	23.1	57.2	35.7	78.6
17	73.3	80	85.7	71.4	35.7	14.3	21.4	66.7	53.3	71.4
18	100.0	50	75	50	25	25	25	100	25	50
19	57.1	42.9	85.7	14.3	14.3	14.3	14.3	42.9	42.9	42.9
20	100.0	83.3	100	33.3	16.7	0	33.3	100	83.3	83.3
21	85.7	85.7	92.9	64.3	35.7	14.3	28.6	57.1	42.8	71.4
22	57.1	100	100	57.1	0	0	0	28.6	71.4	71.4
23	100.0	100	100	50	16.7	33.3	50	50	33.3	33.3
